# Lack of efficacy of psychological and pharmacological treatments of disorders of eating behavior: neurobiological background

**DOI:** 10.1186/s12888-014-0376-7

**Published:** 2014-12-24

**Authors:** Francesca Brambilla, Federico Amianto, Riccardo Dalle Grave, Secondo Fassino

**Affiliations:** The University Department of Psychiatry, San Paolo Hospital, Milano, Italy; University Department of Psychiatry, Torino, Italy; Department of Eating Disorders, Villa Garda, Garda, Italy

**Keywords:** Anorexia nervosa, Bulimia nervosa, Psychotherapy, Pharmacotherapy, Biology

## Abstract

**Background:**

Treatments of eating disorders result too often in partial psychological and physical remission, chronicization, dropout, relapse and death, with no fully known explanations for this failure. In order to clarify this problem, we conducted three studies to identify the biochemical background of cognitive-behavioural psychotherapy (CBT), individual psychology brief psychotherapy (IBPP), and psychotherapy-pharmacotherapy with CBT + olanzapine in anorexics (AN) and bulimics (BN) by measuring the levels of plasma homovanillic acid (HVA) for dopamine secretion, plasma 3-methoxy-4-hydroxy-phenylglycol (MHPG) for noradrenalin secretion, and platelet [3H]-Paroxetin-binding Bmax and Kd for serotonin transporter function. The data were then compared with psychopathological and physical alterations.

**Methods:**

Study 1 investigated the effects of 4 months of CBT on plasma HVA, MHPG and [3H]-Par-binding in 14 AN-restricted, 14 AN-bingeing/purging, and 22 BN inpatients. Study 2 investigated the effects of 4 months of IBPP on plasma HVA in 15 AN and 17 BN outpatients. Study 3 investigated the effect of 3 months of CBT + olanzapine (5 mg/day) in 30 AN outpatients. The data were analyzed using one-way ANOVA for repeated measures for the changes between basal and post-treatment biological and psychological parameters, two-way ANOVA for repeated measures for the differences in the psychobiological data in the 3 groups, Spearman’s test for the correlations between basal and final changes in the psychological and biological scores.

**Results:**

Study 1 revealed significant amelioration of the psychopathology in the AN and BN patients, no effects on HVA, MHPG or Paroxetin binding Kd, and a significant increase in Par-binding Bmax only in the BN patients. Study 2 revealed a significant effect of IBPP on psychopathology in the AN and BN patients, and a significant increase in HVA only in the BN patients. Study 3 revealed a significant positive effect of CBT + olanzapine therapy on the psychopathology and increased HVA values. No correlations were observed in the 3 groups between biological and psychological effects of the three treatments.

**Conclusions:**

Our data advance suggestions on the mechanism of action of the three therapies; however, the lack of correlations between biochemical and psychological effects casts doubt on their significance.

Clinical Trials.gov. Identifier NCT01990755.

## Background

The treatment of Eating Disorders (ED), including anorexia nervosa (AN), bulimia nervosa (BN), and binge eating disorder (BED), is a complex multivariate process which comprises nutritional rehabilitation, psychotherapy, psychopharmacotherapy, and treatment of the medical complications that develop during the course of the disease and that may interfere with its development and prognosis [1-5]. Each treatment is directed at correcting distinct parts of the disorders and each is essential for successful outcome, none being omnivalent or sufficient by itself to induce full recovery, though combined use of multiple therapies is reported to be effective in the treatment of ED. However, they mostly correct the pathological eating behaviour, often leaving out the psychological background which is the likely cause of the diseases. In other words, each treatment takes into consideration a group of symptoms or specific aspects but not the core of the diseases. This may be because the basic cause or the core of ED is still not clear. And in spite of extensive of brain imaging studies identifying the areas pathologically involved in ED [6-8], the brain’s biochemical background behind the onset and course of these disorders has not been clarified, leaving the question open as to which basic therapy could potentially correct it.

Some brain biological alterations present during the course of ED are well known and have been intensively studied. But we still do not know whether they precede these disorders, being their cause, or whether they develop and progress during their course, perhaps subsequent to the nutritional alterations characterizing ED, such as the food deficiencies specific for AN in particular and also in part for BN. What we do know is that the functions of the brain’s serotoninergic, noradrenergic, dopaminergic, acetylcholinergic systems and brain hormonal and neuropeptide secretion are frankly impaired. Yet we do not know if they are only responsible for specific symptomatological aspects of the disorders. [9-14]. Similarly, neuroimaging studies have identified the brain areas whose functions are altered in ED [15-17], mostly in terms of hypo- or hyperfunction, but less is known about the biological meanings of the hypo- or hyper function of these brain nuclei, which raises further questions as to the biochemical impairments that occur with regard to the role neurotransmitters, neurohormones, neuropeptides and most of their receptors in these areas play in the psychopathological alterations related to these syndromes. Hence, the pathogenetic treatment of AN, BN and BED remains a matter of debate. Should DEB be corrected, if possible, by modifying the genetic impairments and the brain’s biochemical alterations that might be the background of the disorders or just by dealing with the full-blown symptomatology of ED?

Up to now, most treatments have included behavioural and physical rehabilitation, taking into consideration correction of the distorted eating attitude but only partially the psychological impairments behind it. Whatever the therapeutic approach, the results are generally insufficient and the recovery partial, followed too often by relapses and chronicization of the diseases.

At this point, a possible validation of specific therapeutic interventions should first include investigation of the brain’s biochemical impairments that have been reported to consistently occur in ED to see whether and how they correlate with the psychopathology of the disorders, and whether they change or disappear during or after treatment, in parallel with specifically correlated psychopathological aspects, ultimately resulting in full contemporary recovery of the psychopathological, behavioural and physical alterations of the diseases.

Following this line of reasoning, we conducted three types of experiments in which we looked at the psychopathological, behavioural and physical aspects of ED and, in parallel, at the concentrations of the peripheral metabolites of noradrenalin and dopamine and at serotonin function, as expressed by the serotonin transporter evaluated by platelet paroxetine binding, before and after psychotherapeutic, psychopharmacological and physical treatments. Our aim was to see whether or not it was possible to find a connection between the brain’s biochemical impairments and specific psychopathological aspects of the diseases, and whether our treatments could correct the brain neurotransmitter alterations in parallel with the psychophysical pathology of the diseases.

## Methods

### Study 1

The first study investigated the effects of nutritional rehabilitation associated with Fairburn transdiagnostic cognitive-behavioural therapy (CBT) [18], as adapted to make it suitable for an inpatient setting by Dalle Grave [19], on the secretion of brain dopamine (DA), noradrenalin (NE) and serotonin (5-HT) in a group of 50 female inpatients, 14 with AN restricted type (AN-R), 14 with the bingeing-purging type (AN-BP), and 22 with BN. Written informed consent for participation in the study was obtained from participants. Patients were all hospitalized at Villa Garda, Garda, Italy. The study was performed in compliance with the Helsinki declaration and the approval of the hospital ethics committee. The brain neurotransmitter functions were examined by peripheral measurement of the main neurotransmitter metabolites: plasma homovanillic acid (HVA) for DA; plasma 3-methoxy-4-hydroxyphenylglycol (MHPG) for NE; and for 5-HT the platelet 5-HT-transporter evaluated by paroxetine binding ([3H ]-Par-binding), divided in Bmax (maximum binding capacity) and Kd (dissociation constant). HVA, MHPG, and Par-binding values have been amply demonstrated to closely mirror the central secretion of the three neurotransmitters and can be used as a correct peripheral measure of neurotransmitter function [20-23]. The aim of the study was to see whether or not NE-DA neurotransmitter metabolites and [3H]-Par-binding are linked to specific aspects of the two diseases, if CBT modifies their secretion, if the physical, behavioural and psychological effects of CBT correlate with changes in neurotransmitter secretions, and if the biological effects of CBT are linked to specific psychopathological aspects of the disorders.

All 50 patients were women. The mean age of patients was 27 ± 7 years in the AN-R group, 22 ± 4 years in the AN-BP group, and 30 ± 8 years in the BN group. The mean duration of disease was 126 ± 50 months in the AN-R group, 120 ± 72 months in the AN-BP group, and 108 ± 60 months in the BN group. The mean body-mass index ([BMI] was 14 ± 1 in the AN-R group, 16 in the AN-BP group, and 19 in the BN group; the mean binging-purging frequency was 10 episodes a week in the AN-BP group and 16 episodes a week in the BN group. All the AN patients were amenorrheic, BN patients were in the follicular phase of the cycle.

The specific psychopathological aspects of the disorders were assessed according to the following instruments: EDE 12-OD, depression by the Beck Depression Inventory (BDI), anxiety by the STAI Form-Y-1, impulsiveness by the Barratt Impulsiveness Scale version 11 (BIS 11), self-esteem by the Rosenberg Self-Biochemical Scale (RSBS), and personality characteristics by the Temperament and Character Inventory-Cloninger Scale (TCI, short form of the 240 items). The biological, physical and psychological measures were assessed at baseline and at the end of 20 weeks of CBT treatment divided into 13 weeks of inpatient treatment followed by 7 weeks of residential day-hospital.

Written informed consent to participate to the study according to the Helsinki declaration was obtained by patients. The study protocol was approved by the institutional Review Board of Villa Garda Hospital, Verona, Italy. The Clinical Trials.gov. Identifier number was NCT01990755.

HVA and MHPG were measured by high-liquid gas chromatography (HPLC) followed by dual-electrode colorimetric electrochemical detection as previously described [24]. [3H] Par binding was measured by spectrometry according to the method of Marazziti et al. [20] as previously described.

The Clinical Trials gov. identifier number was NCT01990755.

### Study 2

This study investigated the effects in 15 AN and 17 BN outpatients of the Psychiatry University Department, Torino-Italy of an individual psychology brief psychotherapy (IBPP) [25] on psychological alterations and DA secretion measured as peripheral blood values of HVA before and after treatment. The study was performed in compliance with the Helsinki declaration, and approved by the university ethics committee.

The mean age of the patients was 20 ± 3.5 years in the AN group and 22 ± 2.8 years in the BN group. The mean duration of disease was 13 ± 8 years in the AN group and 11 ± 4 years in the BN group. The mean BMI was 16 ± 1 in the AN group and 21 ± 2 in the BN group. The duration of IBPP was 4 months. The psychopathological aspects were assessed using the EDI-2, SCL-90, TCI at baseline and after 4 months of therapy. Written informed consent for participation in the study according to the Helsinki declaration was obtained from all subjects. The study protocol was approved by the institutional Review Board of the Psychiatry Department, Turin University, Italy. The Clinical Trials.gov.Identifier number was NCT01990755.

### Study 3

The study evaluated in AN-R and AN-BP patients of the San Paolo Hospital, Milan, Italy, the effects of CBT and of CBT associated with orally administered 5 mg olanzapine on the psychopathological aspects of the disease and on the secretion of HVA to see whether a correlation could be observed between biochemical and psychological disorders typical of ED. The study was performed in compliance with the Helsinki declaration and approved by the hospital ethics committee.

The probands were 18 AN-R and 12 AN-BP patients of the University Dept. of Psychiatry, Milano, Italy. Mean age of the participants was 25 ± 6 years; the mean duration of disease was 5.5 ± 4 years; and the mean BMI was 15 ± 1. All were outpatients and all underwent a 3-month course of CBT, with one half randomly assigned and double-blinded to receive oral olanzapine (2.5 mg for 1 month, 5 mg for 2 months) and the other half an oral placebo. BMI, HVA and psychopathological aspects of the patients were monitored before and at 3 months of therapy by means of the EDI-2, the Hamilton Rating Scale for Depression (HRSD), the Buss-Durkee Rating Scale for aggressiveness (BDRS), the TCI for temperament and character aspects, and the Yale-Brown Cornell for Eating Disorders (YBCS) for obsessivity-compulsivity.

Written informed consent to participate to the study according to the Helsinki declaration was obtained from all subjects. The study protocol was approved by the Institutional Review Board of The Sacco Hospital, Milano, Italy.

The Clinical Trials.gov.Identifier number was NCT01990755.

### Statistical analyses

The data were analyzed using one-way ANOVA for repeated measures to evaluate the significance of the changes in biological and psychological parameters between baseline and post-treatment, and by two-way ANOVA for repeated measures to evaluate the differences in the psychological and biological data in the three groups. The Spearman test was used to evaluate the correlations between basal and final changes in the psychological and biological scores.

## Results

### Study 1

CBT significantly improved the physical aspects of the diseases, particularly BMI in the AN-R and AN-BP patients, and significantly reduced or totally interrupted binging-purging frequency in the AN-BP and BN patients. Psychological assessment revealed that at the end of treatment the ED symptoms, depression, anxiety, impulsiveness and self-esteem improved across all three groups, but more significantly so in the BN than in the AN group. The basal (T0) and final (T1) HVA and MHPG values (Table 1) did not differ significantly in the three groups and decreased but not significantly in all three groups after the end of CBT. Basal and final Par-binding Bmax values did not differ significantly in the three groups and did not change significantly in the AN-R and AN-BP patients, whereas a significant increase was observed in the BN patients. Basal and final Par-binding Kd did not differ significantly in the three groups, and no significant changes were observed in any of the three groups between basal and final values. Basal and final psychological and biochemical data did not correlate. When the difference (Δ) between the basal and final values of neurotransmitter and psychological scores was taken into consideration, no significant correlations were observed between the psychological and the biochemical data.

**Table 1:** **Effects of CBT on HVA, MHPG and [3H] paroxetine-binding values in AN and BN patients**

	**AN-R**	**AN-BP**	**BN**
**HVA (ng/ml)**			
**basal**	17.4 ± 9.0	16.6 ± 8.8	15.6 ± 10.2
**after therapy**	15.6 ± 9.8	12.7 ± 6.0	14.1 ± 9.6
**MHPG (ng/ml)**			
**basal**	10.4 ± 27.1	5.3 ± 4.0	4.4 ± 1.9
**after therapy**	4.4 ± 4.2	4.1 ± 3.8	3.4 ± 1.7
**[3H]-PAR-Bmax (fmol/**			
**mg protein) basal**	446 ± 100	512 ± 175	432 ± 133
**after therapy**	602 ± 161	551 ± 306	645 ± 121*
**[3H]-PAR-Kd (nmol/L)**			
**basal**	0.34 ± 0.48	0.31 ± 0.43	0.26 ± 0.34
**after therapy**	0.24 ± 0.38	0.64 ± 0.14	0.05 ± o.004

Plus-minus values are the means ± SD.

*p = 0.04, r = 1.

### Study 2

At the end of treatment with IBPP the BMI increased significantly over baseline in the AN patients (16 ± 1 vs. 21 ± 2, P <0.001) but was unchanged in the BN patients. Bingeing-purging frequency was consistently reduced in both the AN and BN groups. Psychological impairments after therapy improved in both the AN and BN groups. With regard to the biochemical data (Table 2), HVA values before therapy did not differ in the AN and BN patients and did not change after treatment in the AN patients but increased significantly in the BN patients. No significant correlations were observed between the psychometric test scores and the HVA values before and after therapy, nor between the changes in the two parameters between the two time points, except for some temperamental aspects whose changes showed a positive correlation with those of the DA metabolite, as represented by reward dependence in particular (P <0.02) in both the AN and BN patients, harm avoidance in the AN (P <0.04) and BN patients (P <0.008), self-directedness in the BN patients (P <0.04), self-transcendence in the BN patients (P <0.007) and cooperativeness in the AN and BN patients (P <0.06).

**Table 2:** **Effects of IBPP therapy on HVA secretion in AN and BN patients**

	**AN**	**BN**
**basal**	6.9 ± 2.3	8.1 ± 1,8
**after therapy**	7.6 ± 1.6	11,3 ± 3.3 *

*p < 0.000.

Plus-minus values are the means ± SD.

### Study 3

At the end of 3 months of treatment with CBT and olanzapine (Table 3), the BMI increased consistently in the AN patients without a significant difference between the CBT and the CBT + olanzapine-treated patients (16.2 ± 1 vs. 21.4 ± 2.3; P >0.001). Bingeing-purging frequency was also reduced. The psychopathological symptoms including most of the specific aspects related to eating pathology of the disease improved consistently with both treatments, albeit without significant differences between the two groups, while olanzapine administration significantly improved obsessivity-compulsivity, depression, anxiety and especially hostility (Table 3). HVA blood levels increased significantly in the CBT + olanzapine group, and were unchanged in the CBT group. No correlations were observed between HVA concentrations and the psychopathological parameters examined before and after therapy, nor the changes between the two parameters.

**Table 3:** **Effect of CBT + olanzapine or placebo on HVA secretion in anorexia nervosa**

**patients**	**T0**	**T 1**	**p**	**F**	**d.f**
**Olanzapine**	0.4 ± 0.3	1.1 ± 1.5	0.04	3.6	3
**Placebo**	0.4 ± 0.1	0.3 ± 0.1	NS		

Plus-minus values are the means ± SD.

## Discussion

Even though preliminary the results of our three studies are intriguing. In each of them the psychopathology and the behavioural alterations were significantly improved by CBT or IBPP in the bulimics and somewhat less in the anorexics, while olanzapine treatment seemed to act significantly mostly on comorbid depression, anxiety, obsessivity-compulsivity and hostility, suggesting that a dopaminergic deficiency could be the background of comorbidities more than of a specific ED symptomatology.

However, the effects of the three treatments on NE, DA and 5-HT functions are difficult to interpret. The HVA and MHPG values in Study 1 did not change under CBT in either the AN or BN patients, suggesting that the therapeutic effects of this type of psychotherapy are probably not related to catecholamine function, and that the observed improvements in the altered nutritional behaviour and related psychopathology may not be linked to increases in previously deficient NE and DA secretions. This was suggested also by the observation that when the basal and post-treatment neurotransmitter Δ-values and Δ-psychological scores were correlated no significant correlations were observed between Δ-HVA or Δ-MHPG and Δ-psychopathological aspects of the diseases. With regard to [3H]-Paroxetine-binding Bmax and Kd, the observation that CBT increased 5-HT transporter maximum binding capacity in the BN but not in the AN patients is interesting, since impaired 5-HT brain function has been reported in both AN and BN patients but only BN patients have been reported to show a clear-cut psychopathological recovery after psychotherapy [5,23,26-29], which could be related to the 5-HT transporter improvement we observed. The fact that we could not find specific significant correlations between improvement in 5-HT function and a significant reduction in ED psychopathology in the bulimics, however, seems to exclude that the positive psychophysical effects of CBT are linked to a global correction of 5-HT function.

Two points seem to contradict the correction secretory hypothesis. First, the Par-binding improvement is incomplete regarding the binding capacity of the transporter but not its dissociation constant, thus casting doubt on a global regulatory effect of CBT on 5-HT. Second, the lack of a correlation between the Par-binding-Bmax increase and improvement in BN-specific psychopathology of our patients would suggest that the two improvements, biological and psychopathological, are not linked to one another and therefore that the BN-specific psychological alterations are not linked to a serotonin deficiency. It should be remarked that the rating scales we used to measure the psychopathology of our patients may have missed important aspects of BN and that correlations between 5-HT secretion and clinical symptoms might have appeared if other aspects of the disorder had been taken into consideration. The same observation holds for the type of assay of 5-HT function we used, since Par-binding measures the function of 5-HT transporter and not the direct secretion of 5-HT and its metabolites, 5-HT receptors density and function. All these points should be considered and investigated to evaluate the meaning of our results.

The data from Study 2 are also intriguing. While IBPP was frankly effective on the psychological and behavioural alterations of both AN and BN patients, no changes were observed in HVA secretion in the AN patients, while increased secretion of the DA metabolite was observed in the BN patients. Again, no correlations between the biochemical and psychological aspects of the disorders were observed in either group, except for temperamental characters whose changes under the IBPP therapy revealed a positive correlation with the increase in HVA in both groups. Hence, it may be conjectured that a DA deficiency in AN and BN is not involved in their specific psychological and behavioural pathologies, being instead the possible background of the temperamental alterations which, on the other hand, have been suggested as one of the basic components in ED etiopathogenesis. Obviously, the same objections to our methodology reported for Study 1 can be raised here too. In both studies we used a large variety of rating scales to investigate the entire spectrum of the psychopathological aspects of AN and BN, including the premorbid temperamental characters of the patients, and the psychological, behavioural and comorbid aspects of the full-blown AN and BN pathologies. This does not exclude that our investigation might have missed other psychopathological aspects more significantly improved by IBPP treatment and possibly better correlating with a DA-dependent psychopathology. Moreover, HVA is a mirror of DA secretion but not of DA pre- and postsynaptic receptor activity, which means that we cannot exclude an effect of IBPP on DA system function and aspects we did not take into consideration.

The results of Study 3 are even more intriguing. While CBT significantly improved the specific psychopathology of our patients, olanzapine administration seemed to act mostly on the associated comorbidities like depression, anxiety, hostility and obsessivity-complsivity. Again, a lack of correlation between increased DA activity and decreased AN-specific and unspecific psychopathology seems to exclude the possibility that a DA dysfunction in AN is involved in the pathogenesis of the disorder and its possible recovery. Altered DA secretion has been suggested to be involved in the etiopatogenesis of ED symptomatology [6,7,9,30-34]. However, specific treatments in the past with classical psychotropic drugs acting on DA secretion and receptor functions have not yielded positive results [5,35-37]. More recently, atypical psychotropic drugs including olanzapine, quetiapine, risperidol, clozapine, aripiprazole have been administered [5,38], again, with inconsistent effects. In these therapeutic approaches, drug absorption and its effects on manipulating DA secretion and DA receptor response were not controlled. Study 3 demonstrated that while olanzapine does stimulate DA secretion it does not seem to influence AN-specific psychopathology. But as we did not investigate its pre- and postsynaptic receptor function, we cannot exclude that the lack of efficacy of the drug was not due to postsynaptic receptor insensitivity.

Limits of our studies include the relatively small samples of patients examined, which reduces the generalization of the results and did not permit the subgrouping of the samples in the diagnostic subtypes. A second limit is represented by the choice of running each experiment in different centres. This was due to the necessity of finding in a relatively short time a sufficient number of patients with a perfectly well defined diagnosis.

## Conclusions

At this point we should admit that our approach did not fully elucidate the biochemical impairments potentially underlying ED nor did it further our understanding of the background and significance of the effects of our therapeutic approaches. In particular, we were unable to define clear-cut correlations between the therapeutically-induced changes of basal NE, DA and 5-HT metabolites and of the specific psychopathological aspects of the two disorders. In brief, our protocol of investigation did not help us to better define the brain biochemical background of ED therapies or provide convincing evidence for which type of therapy to suggest. Possibly, other biochemical alterations should be examined in parallel, including hormones, peptides, multiple biochemical parameters expressions of the brain metabolic function together with anatomical alterations of specific brain centers. In other words, a protocol of investigation including the relationship between biochemical and psychological aspects of the patients should be always at the basis of the diagnostic and therapeutic approaches to ED.
